# Acupuncture and rehabilitation of the painful shoulder: study protocol of an ongoing multicentre randomised controlled clinical trial [ISRCTN28687220]

**DOI:** 10.1186/1472-6882-5-19

**Published:** 2005-10-14

**Authors:** Jorge Vas, Emilio Perea-Milla, Camila Mendez, Antonia Herrera Galante, Fernando Madrazo, Ivan Medina, Caridad Ortega, Victoria Olmo, Francisco Perez Fernandez, Luz Hernandez, Jose Maria Seminario, Mauricio Brioso, Francisco Luna, Isabel Gordo, Ana Maria Godoy, Carmen Jimenez, Manuel Anselmo Ruiz, Joaquin Montes, Alonso Hidalgo, Rosa Gonzalez-Quevedo, Pablo Bosch, Antonio Vazquez, Juan Vicente Lozano

**Affiliations:** 1 Unidad de Tratamiento del Dolor, Centro de Salud Dos Hermanas "A", Segovia s/n, 41700 Dos Hermanas, Spain; 2 Unidad de Apoyo a la Investigación (Red IRYSS), Hospital Costa del Sol, Ctra Nacional 340, km 187, 29600 Marbella. Spain; 3 Servicio de Coordinación de Procesos Asistenciales, Subdirección Asistencial-Servicio Andaluz de Salud, Avenida de la Constitución 18, 41001 Sevilla, Spain; 4 Servicio de Rehabilitación, Complejo Hospitalario Carlos Haya, Avda Dr Galves Ginachero s/n, 29009 Málaga. Spain; 5 Servicio de Rehabilitación, Hospital Valme, Carretera de Cádiz s/n, 41014 Sevilla. Spain; 6 Servicio de Rehabilitación, Hospital Infanta Elena. Ctra. Sevilla-Huelva s/n, 21080 Huelva. Spain; 7 Servicio de Rehabilitación, Hospital Infanta Margarita. Avda. de Góngora s/n, 14940 Cabra. Spain; 8 Servicio de Rehabilitación, Hospital General Básico de la Defensa, Carretera de Tentegorra s/n, 30071 Cartagena. Spain; 9 Servicio de Anestesia y Reanimación, Hospital Serranía, Ctra. El Burgo km 1, 29400 Ronda. Spain; 10 Servicio de Rehabilitación, Hospital Serranía, Ctra. El Burgo km 1, 29400 Ronda. Spain; 11 Servicio de Rehabilitación, Hospital Morales Meseguer, Marqués de los Vélez s/n, 30008 Murcia. Spain

## Abstract

**Background:**

Although the painful shoulder is one of the most common dysfunctions of the locomotor apparatus, and is frequently treated both at primary healthcare centres and by specialists, little evidence has been reported to support or refute the effectiveness of the treatments most commonly applied. According to the bibliography reviewed, physiotherapy, which is the most common action taken to alleviate this problem, has not yet been proven to be effective, because of the small size of sample groups and the lack of methodological rigor in the papers published on the subject. No reviews have been made to assess the effectiveness of acupuncture in treating this complaint, but in recent years controlled randomised studies have been made and these demonstrate an increasing use of acupuncture to treat pathologies of the soft tissues of the shoulder. In this study, we seek to evaluate the effectiveness of physiotherapy applied jointly with acupuncture, compared with physiotherapy applied with a TENS-placebo, in the treatment of painful shoulder caused by subacromial syndrome (rotator cuff tendinitis and subacromial bursitis).

**Methods/design:**

Randomised controlled multicentre study with blind evaluation by an independent observer and blind, independent analysis. A study will be made of 465 patients referred to the rehabilitation services at participating healthcare centres, belonging to the regional public health systems of Andalusia and Murcia, these patients presenting symptoms of painful shoulder and a diagnosis of subacromial syndrome (rotator cuff tendinitis and subacromial bursitis). The patients will be randomised into two groups: 1) experimental (acupuncture + physiotherapy); 2) control (TENS-placebo + physiotherapy); the administration of rescue medication will also be allowed. The treatment period will have a duration of three weeks. The main result variable will be the change produced on Constant's Shoulder Function Assessment (SFA) Scale; as secondary variables, we will record the changes in diurnal pain intensity on a visual analogue scale (VAS), nocturnal pain intensity on the VAS, doses of non-steroid anti-inflammatory drugs (NSAIDs) taken during the study period, credibility scale for the treatment, degree of improvement perceived by the patient and degree of improvement perceived by the evaluator. A follow up examination will be made at 3, 6 and 12 months after the study period has ended. Two types of population will be considered for analysis: per protocol and per intention to treat.

**Discussion:**

The discussion will take into account the limitations of the study, together with considerations such as the choice of a simple, safe method to treat this shoulder complaint, the choice of the control group, and the blinding of the patients, evaluators and those responsible for carrying out the final analysis.

## Background

Painful shoulder is one of the most common complaints affecting the locomotor apparatus, and is frequently attended both at primary healthcare centres and by specialists. The annual incidence at primary healthcare centres is 1.2% [1,2]. This pathology, which becomes more common with age [3] and with the practice of certain occupations and sports, is evidenced mainly by pain, restricted movement and strength and by the loss of shoulder functionality. The incidence on occupational invalidity, though mentioned by most authors, remains unknown; only approximate data are available, such as those provided by *Instituto de Biomecánica *at Valencia, which has estimated that 50% of sick leave is accounted for by muscle or bone injuries in the shoulder or neck.

The most common actions taken at present to relieve the symptoms of painful shoulder include steroid injections, physiotherapy, oral NSAIDs and "wait and see"; hardly any of these measures have been tested scientifically to demonstrate their effectiveness in this situation [4]. According to the bibliography reviewed, physiotherapy, which is the most common treatment applied in this case, has not been conclusively shown to be effective; sample sizes have been small and methodological rigor lacking from the papers published in this respect. On the other hand, solid evidence has been produced that ultrasound therapy is ineffective in treating the painful shoulder [1,5,6].

The most relevant bibliography we have examined comprises reviews of the question carried out by the Cochrane group in 2000, 2003 and 2004, together with certain systematic reviews by other authors. In general, the conclusions of the analysts in the Cochrane studies, and of other reviewers working for professional organisations, is that there is very little evidence to support or refute the effectiveness of the treatment commonly applied for the painful shoulder. Therefore, well-designed studies are necessary, using uniform methods of diagnosis and measurement to ensure the validity of the results obtained. We have also analysed other studies, with less backing than the above-mentioned opinions of expert reviewers, but with an acceptable methodology, that have concluded in favour of one or other mode of treatment, such as physiotherapy [7,8] or the injection of corticoids [9,10]. We have also examined the recommendations of panels of experts and members of associations of physiotherapists and GPs.

Acupuncture has been used to treat this type of medical problem in China for over 3000 years. At present, it is under consideration as a technique to be applied in Western medical practice for a great many complaints, especially for cases in which modern techniques are either of limited effectiveness or are unsuitable [11]. Acupuncture is now widely used in the treatment of chronic pain [12-14]. The systematic review carried out by Lewith & Machin on the effectiveness of acupuncture in treating chronic pain concluded that treatment with "real" acupuncture was significantly more effective than that with "false" acupuncture and with a placebo [15]. It has also been shown that acupuncture provokes fewer adverse side effects than does the use of NSAIDs or opiates [16]. In recent years, randomised controlled studies have provided further evidence supporting the use of acupuncture in the treatment of pathologies of the soft tissues of the shoulder. For example, Kleinhenz et al. [17] observed an improvement of 19.2 points on Constant's scale for an experimental group versus one of 8.37 points among a control group, using a placebo featuring retractable needles, but these authors seem to have focused more on demonstrating the effectiveness of the technique used with the control group than on the specificity of the selection of the acupuncture points; moreover, with respect to the analysis of the main result variable (the absolute improvement achieved), the fit to the baseline measurement was not made. On the other hand, Sun et al. [18] (did aim to locate the specificity of the points, by selecting a point that was distal from the affected area, using a randomised controlled test with two groups; 13 patients were treated with acupuncture and specific exercises for the shoulder, and another 22 patients were treated solely with exercise. The design of the latter study is similar to that presented in this project although we believe the sample size is too small and a significant degree of bias was introduced by the fact that the control group (which received only exercises) did not receive the same medical attention as the experimental group. Nevertheless, the results obtained with the experimental group were significantly better than those of the control group. A further problem is that the point proposed by the authors (Zhongpin of the leg) is difficult to locate, as its situation is not constant, on the contrary to that of Tiaokou (ST38), which is situated exactly 8 cun below the articular line of the knee and 1 cun lateral from the tibial crest. Another clinical trial was reported by Gilbertson et al. [19], who described a case in which, after an arthroscopic intervention on the shoulder, traditional and sham acupuncture were compared. It was concluded that real acupuncture provides a significant improvement, concerning the degree of analgesia achieved, the reduction in the quantity of analgesics required, increased mobility and patient satisfaction; however, the acupuncture points selected are not described and so the trial is not reproducible.

Since the introduction of acupuncture techniques into primary healthcare at the *Dos Hermanas "A" *Health Centre, with the establishment of the Pain Treatment Unit, data have been compiled to obtain an initial evaluation of the reactions of patients who are given acupuncture treatment [20]. Moreover, a pilot study has been carried out to assess the immediate effects of this technique when applied to cases of supraspinal tendinitis [21] as a previous step to the development of the present study.

In this article, we describe a randomised, blinded, multicentre study carried out with a sufficiently-large sample group, with systematised, uniform diagnostic criteria, homogeneous therapeutic interventions including the use of a placebo for the control group, follow up periods exceeding three months and validated measurement of results. We believe such a systematic approach is necessary to clearly describe the current context of treatment for the painful shoulder. In the study, we work on the hypothesis that the acupuncture of Tiaokou ST38, together with physiotherapy, can reduce pain and improve functionality in situations of subacromial syndrome (rotator cuff tendinitis and subacromial bursitis) to a greater extent than does physiotherapy associated with a TENS-placebo treatment. The study began in March 2005 and the recruitment phase remains open.

## Methods/design

### Design

Randomised controlled multicentre study with blind evaluation by an independent observer and blind, independent analysis.

### Study subjects

Patients referred to the Rehabilitation services of the health centres participating in the study. These centres are part of the public health systems of the regions of Andalusia and Murcia (Spain). The patients presented chronic symptoms of subacromial syndrome (rotator cuff tendinitis and subacromial bursitis) and were offered treatment with physiotherapy together with acupuncture or transcutaneous stimulus. They were informed of the characteristics of the study and of the techniques to be used, as well as of the possible risks (infection, lipothymia, hematomas). They were told they could leave the study at any moment, with no type of penalisation or loss of benefits to which they were entitled.

### Selection criteria

Inclusion criteria

• Patients with a clinical diagnosis of subacromial syndrome (rotator cuff tendinitis and subacromial bursitis) with a case history > 3 months

• Prior radiography, with normal results

• Informed consent

• Unilateral injury

Exclusion criteria: surgery, luxations or fractures in the proximity of the shoulder; other severe direct or indirect traumas (in traction) observed in the anamnesis and clearly related to the onset of the current episode; hypocoagulates, generalised disorders of the musculoskeletal system or neurologic disorders, vascular trophic disorders in the lower limbs, lymphedema.

### Ethical criteria

The ethical validity of this study has been analysed and approved by the corresponding ethical and research committees at the healthcare centres involved. The study design takes into account the Principalism criteria of Beauchamp & Childress (beneficence, non-maleficence, autonomy and justice) and expressly guarantees the patient's right to privacy and informed decision-making. The study also complies with the norms for Good Clinical Practice and the Edinburgh 2000 revision of the Helsinki Declaration. All the patients who participate give their written, informed consent to the clinical research methods applied. During the development of the study, audits will be performed, according to the criteria of the Research and Ethics Committee and the healthcare centre's Quality Committee, independently of the external audits (research funding provider) that may be required.

### Criteria and procedures for withdrawal from the study

A patient may be withdrawn from the study at any time, either at will or by decision of the researcher. The reasons for interrupting participation in the study will be recorded on the summary page of the Digital Data Record (DDR). The following procedure is to be followed when a patient withdraws from the study:

• Assess the relevant study variables

• Record any adverse events

• Evaluate the taking of rescue medication

• Indicate the possible co-interventions carried out

• Complete the DDR, record the date and reason for withdrawal.

### Randomisation

The patients will be assigned on a random basis to the two study groups: 1) experimental group treated with acupuncture plus physiotherapy; 2) control group, to be given the TENS placebo plus physiotherapy. Randomisation will be carried out in each digital data recording system. Every healthcare centre participating in the study has a specially-designed DDR based on a Dell Axim × 30 PDA; once a new patient's data are entered, the patient is randomly assigned a treatment code (A = experimental; B = control). This code is concealed from the evaluator. Each PDA system has three access codes, one for the study controller, one for the evaluator and one for the doctor carrying out the treatment, and only the latter has access to the treatment code. The research team will take the necessary measures to ensure the confidentiality of the patients taking part, including their deidentification within the databases constructed for the analysis.

### Interventions

(See Figure 1)

### Acupuncture (experimental group): 3 sessions (once weekly)

Once a week, before the physiotherapy session, the doctors responsible for the treatment (specialists who are well-acquainted with the technique) will apply acupuncture at the Tiaokou ST38 point, following the *tiao-shan *homolateral technique. This consists of the perpendicular insertion of a single-use sterile filiform acupuncture needle, 7.5 cm long, 30 gauge body diameter, using a guide-tube. The insertion is to be made, after sterilising the skin and with the patient in a prone position, at the Tiaokou point (located equidistally from the flexion fold of the knee and the vertex of the lateral malleolus and 1 inch laterally from the tibial crest, to a depth of 4.5 – 5.0 cm, towards the Chengshan UB57 point (located on the rear surface of the leg, half way between the popliteo fold and the heel, in an inverted-V shaped crease separating the cords of the external calf. Insertion of the needle is followed by vigorous stimulation by means of broad bidirectional rotation movements of the body of the needle, intended to produce the sensation known as Deqi, often described as one of irradiance. The needle is maintained in place for 20 minutes, and manipulated for 1 minute every 5 minutes (i.e. 4 manipulations per session). While the needle is being manipulated, the patient should perform abduction and external and internal rotation exercises.

The single-use sterile needles are manufactured by Cloud & Dragaon Radical Device Co., Ltd (Wujiang, China), according to EU norms, and are imported by Acupuncture Shop, Storegade 58, 6800 Vade (Denmark).

### TENS placebo (control group): three sessions (once weekly)

Once a week, before the physiotherapy session, the doctors responsible for the treatment will apply the TENS placebo, which consists of placing two adhesive electrodes, one each on the front and rear surfaces of the leg that is homolateral to the affected shoulder. The electrodes are connected to a deactivated TENS apparatus, model 8016 M. The stimulation unit remains placed in front of the patient, such that the flashing of the diode simulating the stimulus is visible at all times. The patient remains in the same position for 20 minutes, after which the TENS unit is disconnected and the electrodes removed from the patient's body. The frequency of the sessions is the same as that for acupuncture.

### Physiotherapy (experimental and control groups): 15 sessions (3 weeks)

The physiotherapy sessions last 40 minutes each and consist of the following (see Additional file 1 1 – Physiotherapy protocol [ISRCTN28687220]):

• Superficial heat therapy (graduated according to the patient's sensations; 5 minutes)

• Recentering of the humeral head (active manoeuvres: 5 minutes; passive manoeuvres: 5 minutes)

• Diadynamic currents, diphase attached with positive pole at the point of greatest pain (graduated according to the patient's sensations; 5 minutes)

• Post-session cryotherapy (10 minutes).

The patient is recommended to avoid any activity that may cause pain in the affected arm for the duration of the study. Ultrasound therapy was not included as an applicable technique because it has been shown to be ineffective in treating pathologies in this region [4].

The daily physiotherapy sessions will follow those of acupuncture and the TENS placebo in the following way: first, a session of acupuncture or TENS placebo followed by the first one of physiotherapy; the next four sessions of physiotherapy to be applied on following consecutive days. The second acupuncture or TENS placebo session is applied prior to the sixth one of physiotherapy, and is followed by another four physiotherapy sessions during the next four consecutive days. The third and final acupuncture or TENS placebo session is applied prior to the eleventh one of physiotherapy, and is followed by another four physiotherapy sessions during the next four consecutive days, after which the final assessment is made. The acupuncture and the TENS placebo sessions take place in identical rooms.

### Rescue medication

The patients are allowed to take analgesics and/or NSAIDs if they wish. If they do, this fact, and the daily dose taken, should be recorded in the digital data record (DDR).

• Anti-inflammatory medication

• If taken, the maximum dose allowed is 1 diclophenac pill (50 mg) three times a day for a maximum of 4 weeks.

• Administration instructions: to be taken with meals, to alleviate possible gastric irritation.

• Gastroprotective medication

• Specific indications:

• No past record of ulcers or risk factors such as anticoagulant therapy, association with corticoids, age > 60 years or severe baseline illness (kidney insufficiency, cirrhosis, COPD): NO GASTROPROTECTION

• No past record of ulcers and risk factors such as anticoagulant therapy, association with corticoids, age > 60 years or severe baseline illness (kidney insufficiency, cirrhosis, COPD): GASTROPROTECTION with 200 μg misoprostol every 6 or 12 hours or 20 mg famitidine every 12 hours.

• Past record of ulcers: GASTROPROTECTION with 20 mg omeprazole every 24 hours.

• Contraindications: diclophenac should not be taken by patients allergic to it, to acetylsalicylic acid or other NSAIDs, by patients with a history of asthma, angiodema or rhinitis provoked by NSAIDs, by patients affected by porphyria or with a history of ulcers, coagulation pathologies or haemorrhages. Caution is advised for patients with kidney insufficiency, cardiac insufficiency, hypertension or liver insufficiency.

### Study variables

#### Baseline assessment (T-0)

For the sake of consistency, criteria of selective tension and mobility patterns [22]that we believe are clear enough for homogeneous diagnoses to be achieved (see Additional file 2) will be applied. When the patient has been diagnosed, he/she will be invited to give informed consent to take part in the study. If this is received, the initial assessment will be made by an external rehabilitation evaluator, who will record the following data:

• Sociodemographic data

• Age

• Sex

• Influence on the shoulder of the type of work performed (no effect, moderate effect, severe effect)

• Background

• Previous episodes of shoulder pain (number of episodes)

• If previous episodes occurred, did they provoke sick leave? (No. of such events and their duration)

• Current episode

• Concomitant neck pain(yes/no)

• Duration of the present episode (in months)

• Previous treatment received for the same episode (corticoid injections, analgesic or anti-inflammatory medication)

• Acute onset of the present episode (yes/no)

• Direct cause (excessive tension or otherwise, slight injury or unknown cause)

• Is the affected shoulder the dominant one? (yes/no)

• Constant's Shoulder Function Assessment (SFA) Scale

• Pain intensity in the shoulder during the day, on a 10 cm Visual Analogue Scale (VAS)

• Pain intensity in the shoulder during the night, on a 10 cm Visual Analogue Scale (VAS)

• Analgesic and NSAID medication taken during the previous 2 weeks (4 point Likert scale: 0 no medication; 1 less than the usual daily dose; 2 the normal dose; 3 more than the normal dose).

### Main result variable (endpoint)

At 3 weeks (T-1) after the start of the study, the results of the intervention will be assessed by an independent expert evaluator. The 3-month follow up will be performed by the same evaluator. An additional follow up will be carried out at 6 and 12 months after the final evaluation (by independent telephone interviewer), including the main result measurements. These are the change in the SFA score, with respect to baseline values, after the 15th physiotherapy session. This assessment will be made by an external rehabilitation specialist with no information regarding the treatment received by the patient. Constant's Shoulder Function Assessment (SFA) Scale has a maximum score of 100 points, including subjective and objective elements in a proportion of 35/65, respectively. The subjective parameters describe the degree of pain felt by the patient and his/her ability to carry out normal daily activities, as regards both the level of activity and the position of the arm. The objective parameters are based on the range of active compound movements that enable the arm to be moved to functionally relevant positions, using a goniometer to measure the rear and lateral elevation and the positioning of the hand in relation to the head and the trunk in order to assess the degree of rotation achieved. The score for the power exerted by the shoulder is based on the weight (in kg) the patient can raise in abduction, to a maximum of 11 kg. A total SFA score of 100 indicates a shoulder with perfect freedom of movement, no pain and normal functioning.

### Secondary variables

After the first week of treatment, the level of confidence in the treatment is measured on a Treatment Credibility Scale (TCS) [23]. This was first proposed by Borkovec and Nau [24] and comprised four items that are assessed on a continuous VAS from 0 to 10 (0 totally disagree; 10 totally agree). The following elements are included, but at the baseline interview, only the first two questions are asked:

1. Are you confident this treatment will alleviate the pain you feel?

2. Does the treatment seem a logical one?

3. Would you recommend this treatment to a friend or relative who had the same problem?

4. Do you think this treatment could be applied to other problems?

After three weeks of treatment (T-1) (15 sessions of physiotherapy), the following secondary variables will be assessed by an independent evaluator:

• Pain intensity in the shoulder during the day, on a 10 cm VAS (DPI-VAS)

• Pain intensity in the shoulder during the night, on a 10 cm VAS (NPI-VAS)

• Degree of improvement perceived by the patient (IPP) (7 point Likert categoric scale: 0 much worse; 1 worse; 2 slightly worse; 3 no change; 4 slightly better; 5 better; 6 much better) [25]

• Degree of improvement perceived by the evaluator (IPE) (7 point Likert categoric scale: 0 much worse; 1 worse; 2 slightly worse; 3 no change; 4 slightly better; 5 better; 6 much better)

• NSAIDs taken (NT)

• Treatment Credibility Scale items 3 and 4

• Adverse events (ADEV) of a traumatic nature that might distort the results of the evaluation.

#### Follow up at 3 months after completing the treatment (T-2)

An independent evaluator and rehabilitation specialist will make the following assessments at 3 months after the treatment ends:

• Score on Constant's Shoulder Function Assessment (SFA) Scale

• Pain intensity in the shoulder during the day, on a VAS (DPI-VAS)

• Pain intensity in the shoulder during the night, on a VAS (NPI-VAS)

• Degree of improvement perceived by the patient (IPP)

• Degree of improvement perceived by the evaluator (IPE)

• NSAIDs taken (NT)

• Adverse events (ADEV)

• Subsequent episodes of shoulder pain, their duration and treatment applied.

#### Follow up at 6 months after completing the treatment, carried out by telephone interview (T-3)

An independent evaluator will make the following assessments by telephone interview at 6 months after the treatment ends:

• Score on Constant's Shoulder Function Assessment (SFA) Scale (only the subjective parameters)

• Pain intensity in the shoulder during the day, on a VAS (DPI-VAS)

• Pain intensity in the shoulder during the night, on a VAS (NPI-VAS)

• Degree of improvement perceived by the patient (IPP)

• NSAIDs taken (NT)

• Adverse events (ADEV)

• Subsequent episodes of shoulder pain, their duration and treatment applied.

#### Follow up at 12 months after completing the treatment, carried out by telephone interview (T-4)

An independent evaluator will make the following assessments by telephone interview at 12 months after the treatment ends:

• Score on Constant's Shoulder Function Assessment (SFA) Scale (only the subjective parameters)

• Pain intensity in the shoulder during the day, on a VAS (DPI-VAS)

• Pain intensity in the shoulder during the night, on a VAS (NPI-VAS)

• Degree of improvement perceived by the patient (IPP)

• NSAIDs taken (NT)

• Adverse events (ADEV)

• Subsequent episodes of shoulder pain, their duration and treatment applied.

### Data collection and analysis

Data collection and recording will be carried out using an Dell Axim × 30 PDA, fitted with the Windows^® ^operating system, and specially designed for the purposes of this study, with obligatory fields, validation rules and quality control for error avoidance; a 512 bit encoding system is incorporated to ensure the confidentiality of all the records. Furthermore, as additional functions, it will include a security analysis system with stored passwords, and functions for the export/import and synchronisation of the database content. Access to this PDA will be limited to THREE users: 1) the healthcare centre evaluator; 2) the doctor applying the treatments; 3) the study controller. Each will have a different access code.

The data tables describing the characteristics of the study subjects and the results of each of the evaluations will be entered separately. Each record corresponding to an evaluation, after approval by the researcher responsible, will be stored such that further editing cannot be performed. A backup copy of the database will be encrypted in a 512 bit system and uploaded weekly by safe transmission system to a web server. The study controller will store on the central computer the data obtained from each of the participating healthcare centres and will include all the records in the central database. Similarly, the study controller will make a weekly printout of the updated tables from each of the healthcare centres; these documents will be stored securely and kept as an original record for purposes of inspection or auditing or in case of loss of digital data. Each PDA will be the responsibility of the corresponding researcher at each health centre.

### Sample size and associated power

The sample size was predetermined for a level of significance of 0.05, a power of 0.80 and a final average score on Constant's SFA scale of 70 among the experimental group (standard deviation 17) and 65 among the control group (standard deviation 18), with a two-tailed test, according to data taken from Kleinhenz [17]and Sun [18]. Thus, 188 patients are required for the experimental group and 199 for the control group. Assuming a 20% dropout rate, the final sample size was set at 226 patients for the experimental group and 239 for the control group [26].

### Population

For the purposes of the analysis, two types of population are to be considered:

• Per intention to treat (ITT): this population will consist of all the randomised patients. Those taking part in the selection phase but not subsequently randomised for treatment will be excluded from this population. This will comprise the main population for analysis of the parameters of effectiveness.

• Per protocol: this population will consist of all the patients in the ITT population with no serious deviations from the protocol. This will comprise the secondary population for analysis.

### Treatment comparisons

All the tests will be bilateral and carried out at a level of significance of α = 0.05. So as not to interrupt the study, no intermediate analyses are foreseen.

### Data analysis

To describe the different baseline characteristics of the patients, we shall use measures both of position (the mean or the median, according to the asymmetry) and of variability (standard deviation or interquartile range) for the continuous variables, while the others will be described via their frequency distributions, comparing the experimental and control groups. Moreover, a graphic analysis will be made, using smoothed versions of the histograms, boxes and other representations.

For the principal result variable, the difference between the final value and the baseline Constant SFA score, measured in absolute terms, the experimental and control groups will be compared after adjusting by the baseline value and subsequently by other possible confounders. This analysis will be repeated in the follow ups at 3, 6 and 12 months. We will evaluate the effect of possible points of influence by repeating the model after exclusion of the observations with a large Cook-distance value. The assumptions of the model will be tested and reanalysed using a Q-Q plot diagram for the assumption of normality, and by comparing the studentised residuals versus the values of the independent variable for the assumption of constant variances.

For the secondary result variables, the bivariate analysis will be performed using contingency tables analysed by ji-squared tests (for tables larger than 2 × 2), by ji-squared tests corrected for continuity, or Fisher's exact test (for 2 × 2 tables), for the categoric variables, and by simple linear regression for all other cases. Two-dimensional graphs will also be used.

The multivariate analysis will depend on the nature of the response variable. When this is dichotomic, a binary logistic regression analysis will be made, and for this purpose we will establish the dummy variables needed for the categoric predictors. The inclusion of the "experimental group" or "control group" variable will be forced. The "forward procedure" method will be used to select the possible confounding variables in the model, and the entry criterion used will be the change in the likelihood ratio. The functional form of the continuous predictors will be studied via the corresponding statistical criteria, these being mainly graphic procedures.

For continuous variables, a multiple linear regression model will be used, and the same model-selection criteria and diagnostic procedures as described above will be applied.

### Current status of the trial

Recruiting of the patients began in March 2005 and will continue until December 2005. Follow up is planned to end in March 2007.

## Discussion

We believe that one of the most interesting features of this study is the fact that it introduces a neurostimulative technique, and one that is relatively simple to apply, into rehabilitative medicine. Nevertheless, we are also aware that this comprises one of its greatest difficulties, as this alternative treatment has been very little used, to date, in this field. The possibility of extending this clinical practice into rehabilitation services is one of the challenges we seek to overcome.

It is very difficult to assess all the aspects that go to make up chronic pain, as it is such a subjective experience. This poses problems as regards finding theoretical models and appropriate measuring instruments, although the correct application of experts' recommendations will make it possible to standardise results for later analysis.

There is little evidence to support or refute the effectiveness of the most common interventions to treat the painful shoulder. As well as the necessity to carry out further, well-designed clinical studies, we need to establish a uniform means of defining shoulder pathologies and of developing valid result measurements that are consistent and adaptable to changes in the population. The choice of a single type of pathology prevents us from extrapolating the possible results of research to all the situations in which painful shoulder is diagnosed. However, the fact of selecting a single diagnosis will ensure the specificity of the intervention and will enable us to evaluate particular aspects of the process.

The clinical studies carried out to examine non conventional medical treatments such as acupuncture pose serious problems regarding the study design and possible bias. In selecting the variables to be examined in this study, we have taken into consideration the previous experience of the Pain Treatment Unit at the *Dos Hermanas "A" *Health Centre and on the comments made in the systematic reviews of the field made by the Cochrane Group.

The placebo selected differs considerably from the acupuncture technique under study, but the intervention in itself is, in fact, difficult to disguise [27]. The depth of needle insertion and the manipulations subsequently effected prevent the use of placebos that are totally credible [28]. This is why we decided to apply an inactive control treatment (the TENS placebo), in order to subject the two groups of patients to the same rhythm of intervention. Nevertheless, the degree of credibility of the intervention and of the placebo will be assessed at the start and at the end of the study. This type of control was first used experimentally by Macdonald [29]. The placebo effect is related to the patient's expectation, to the observer and to the doctor's attention, in combination with classical Pavlovian-type response conditioning activated by the positive or negative expectation of a cure. Petrie and Hazleman [30]used a scale to measure the credibility of acupuncture treatment and that of the TENS placebo, and reached the conclusion that both methods were equally credible, thus justifying the use of the TENS placebo as a control.

Although the characteristics of the intervention prevent us from establishing a double blind design, we intend to blind the patients from the evaluator, and believe this simple blinding will make it possible to achieve sufficient control of possible bias.

As with any other multicentre study, we are confronted with certain disadvantages, such as the loss of unity of judgement, with the participation of various researchers, or the reduced homogeneity of the sample group and the parallel increase in data dispersion. In order to overcome these problems, we have had to create very simple criteria for patient selection, to avoid possible subjectivity as concerns the different researchers. Additionally, we have laid great stress on the need to obtain a good level of communication between the different work groups, a condition that is sometimes essential to standardise criteria and to guarantee the internal validity of the study. The best way to achieve this communication is by means of frequent meetings, the coordination of which will be the responsibility of the study controller.

The limited number of patients and the restrictions imposed on the study concerning the criteria for inclusion are counteracted by its extension to healthcare centres serving populations living in a different cultural situation, and to centres that are organised in different ways. Thus, the external validity of the study is safeguarded.

## Abbreviations

COPD: Chronic Obstructive Pulmonary Disease

DDR: Digital data record

GP: General Practitioner

ITT: Intention to treat

NSAIDs: Non-steroid anti-inflammatory drugs

PDA: Personal Digital Assistant

SFA: Shoulder Function Assessment Scale

TCS: Treatment Credibility Scale

TENS: Transcutaneus Electric Nerve Stimulation

VAS: Visual analogue scale

## Competing interests

The author(s) declare that they have no competing interests.

## Authors' contributions

Conception and design: J. Vas. Revision of the different versions of the study protocol: J. Vas, E. Perea-Milla, C. Mendez, A. Herrera Galante, F. Madrazo, I. Medina, C. Ortega, V. Olmo, F. Perez Fernandez, L. Hernandez, J. M. Seminario, M. Brioso, F. Luna. Substantial contributions to the conception and design of the digital data record: I. Gordo, A. M. Godoy, C. Jiménez, M. A. Ruiz, J. Montes, A. Hidalgo, R. Gonzalez-Quevedo, P. Bosch, A. Vazquez, and J. V. Lozano. All authors have read and approved the final manuscript.

The study protocol was developed in 2004 and is co-funded by *Fundación Progreso y Salud *(File No. 82045) from the recruitment phase until the short term evaluation, and by *Consejeria de Salud de la Junta de Andalucia *(File No. 136/04) for the long term follow up phases. The publishing of this article is funded by the IRYSS Network.

## Pre-publication history

The pre-publication history for this paper can be accessed here:



## Supplementary Material

Additional File 1 HD Physiotherapy protocol ISRCTN28687220.doc Additional explicative document of the physiotherapy protocol for the patients included in the study. Click here for file

Additional File 2 HD Table 1 ISRCTN28687220.doc Table 1. Examination protocol Click here for file
