# Supplementary material for: Acupuncture and rehabilitation of the painful shoulder: study protocol of an ongoing multicentre randomised controlled clinical trial [ISRCTN28687220]
Source: BMC Complement Altern Med. 2005 Oct 14;5:19. doi: 10.1186/1472-6882-5-19 (PMC1277817; doi:10.1186/1472-6882-5-19)
Supplement: Additional File 1 — HD Physiotherapy protocol ISRCTN28687220.doc Additional explicative document of the physiotherapy protocol for the patients included in the study. [file 1472-6882-5-19-S1.doc]

# Physiotherapy protocol [ISRCTN28687220]

This protocol is designed for the treatment of subacromial dysfunction, both bursal and tendinous, alike and without individualisations. The main aim of this protocol, common to both cases, is the decompression of the subacromial space by passive and active modes of application. The relief of pain and inflammation is sought by the application of cryotherapy (cold pack), which is known to have analgesic and anti-inflammatory effects.

## Protocol for physiotherapeutic treatment

- Superficial heat therapy**,** by infrared lamp aimed at the anterolateral surface of the shoulder to be treated, placed at a distance of about 15 cm, for a period of 5 minutes.
- Recentering of the humeral head**,** by active and passive manoeuvres aimed at returning the humeral head to its natural position, reestablishing the subacromial space and thus alleviating the compressive action on the subacromial structures.
  - Active manoeuvres**.** The patient, by activating the depressor muculature of the humeral head, produces a downward force to enlarge the subacromial space. The optimum position is that in which the patient is seated, with the shoulder in 50-60º of abduction and with the arm raised to the level of the scapula (about 30º antepulsion), with the elbow resting on a table or couch. A logical procedure would be: 5-6 seconds of sustained lowering followed by 5-6 seconds’ rest, for a total working time of 5 minutes. This activity should never be painful. *Advice:* to clarify the manoeuvre to the patient, it is often useful to suggest he/she moves away from the therapist’s index finger when this is approached to the tubercle. Total time: 5 minutes.
  - Passive manoeuvres**.** These can be carried out in the same position as above, using the first commissure of the mobilising hand, while the other one is placed on the scapula and plays a stabilising role. The direction in which the humeral head should move is vertically downwards, and slightly backwards. Otherwise, and with the same aim in mind, always depending on the criteria of the physiotherapist, the manoeuvre can be carried out with the patient lying prone, with the necessary modification of the directions. All movements should be smooth, rhythmic, repetitive and of small amplitude. Total time: 5 minutes.
- Dynamic control of the scapula**,** by which we seek to reestablish an appropriate scapulo-humeral rhythm, promoting the physiological sequential activation of the periscapular musculature. Basically, it consists of activating, at the right time and in the right way, the musculature that is antagonist to the protraction of scapula, the too-early activation of which alters the scapular-humeral rhythm and tends to provoke subacromial impingement. The patient is told the purpose of this scapular retraction exercise and how best to perform it, by lowering the shoulder blades and actively contracting the lower angle of the scapula. The contraction position should be maintained for 6 seconds. Total time: 5 minutes.
- Post-session cryotherapy, using a cold pack applied to the lateral surface of the shoulder, with the centre of this pack located as close as possible over the cuff tendon. The pack should have been kept at least 4 hours in a freezer at -4 to -8ºC. The frost should be removed and the pack wrapped in a towel or cloth. The duration of the application is 10 minutes.
- Maintaining the gains made and practical advice for everyday activities. The patient is advised how to maintain the results / benefits obtained. The performance of any activity that might worsen the symptoms is advised against. Such activities include raising the arm above the head, lifting and carrying heavy loads, any sporting or occupational activity that makes special, selective demands of the shoulder and inappropriate sleeping positions. On the contrary, the patient is recommended to practise measures and movements that alleviate the symptoms.
