# Supplementary material for: Acupuncture and rehabilitation of the painful shoulder: study protocol of an ongoing multicentre randomised controlled clinical trial [ISRCTN28687220]
Source: BMC Complement Altern Med. 2005 Oct 14;5:19. doi: 10.1186/1472-6882-5-19 (PMC1277817; doi:10.1186/1472-6882-5-19)
Supplement: Additional File 2 — HD Table 1 ISRCTN28687220.doc Table 1. Examination protocol [file 1472-6882-5-19-S2.doc]

Table 1 - Examination protocol

| **Anamnesis**: characteristics of the pain, functional effect, previous episodes, associated symptoms, previous and current treatment, medical history, dominant hand, professional and recreational activities. |
| --- |
| **Physical examination**:  Examination of the cervical spine  Examination of the 12 shoulder movements:  Complete active raising of the arm  Complete passive raising of the arm  Complete active raising of the arm (painful arc)  Passive abduction  Passive external rotation  Passive internal rotation  Resisted abduction of the arm  Resisted approach of the arm  Resisted external rotation of the arm  Resisted internal rotation of the arm  Resisted flexing of the elbow  Resisted extension of the elbow |
| **Complementary tests**: if rupture of the supraspinal tendon is suspected, an echographic examination will be made of the soft tissues to prove/disprove this diagnosis. |
